# Volumetric analysis of the aging auditory pathway using high resolution magnetic resonance histology

**DOI:** 10.3389/fnagi.2022.1034073

**Published:** 2022-11-10

**Authors:** Eric Y. Du, Briana K. Ortega, Yuzuru Ninoyu, Robert W. Williams, Gary P. Cofer, James J. Cook, Kathryn J. Hornburg, Yi Qi, G. Allan Johnson, Rick A. Friedman

**Affiliations:** ^1^Department of Otolaryngology—Head and Neck Surgery, University of California, San Diego, San Diego, CA, United States; ^2^Department of Genetics, Genomics and Informatics, University of Tennessee Health Science Center (UTHSC), Memphis, TN, United States; ^3^Department of Radiology, Center for In Vivo Microscopy, Duke University Medical Center, Durham, NC, United States

**Keywords:** magnetic resonance imaging, aging, neuroanatomy, auditory pathway, neuroaging, volumetric 3D, hearing, BXD family

## Abstract

Numerous shown consequences of age-related hearing loss have been unveiled; however, the relationship of the cortical and subcortical structures of the auditory pathway with aging is not well known. Investigations into neural structure analysis remain sparse due to difficulties of doing so in animal models; however, recent technological advances have been able to achieve a resolution adequate to perform such studies even in the small mouse. We utilize 12 members of the BXD family of recombinant inbred mice and aged separate cohorts. Utilizing novel magnetic resonance histology imaging techniques, we imaged these mice and generated high spatial resolution three dimensional images which were then comprehensively labeled. We completed volumetric analysis of 12 separate regions of interest specific to the auditory pathway brainstem nuclei and cortical areas with focus on the effect of aging upon said structures. Our results showed significant interstrain variation in the age-related effect on structure volume supporting a genetic influence in this interaction. Through multivariable modeling, we observed heterogenous effects of aging between different structures. Six of the 12 regions of interests demonstrated a significant age-related effect. The auditory cortex and ventral cochlear nucleus were found to decrease in volume with age, while the medial division of the medial geniculate nucleus, lateral lemniscus and its nucleus, and the inferior colliculus increased in size with age. Additionally, no sex-based differences were noted, and we observed a negative relationship between auditory cortex volume and mouse weight. This study is one of the first to perform comprehensive magnetic resonance imaging and quantitative analysis in the mouse brain auditory pathway cytoarchitecture, offering both novel insights into the neuroanatomical basis of age-related changes in hearing as well as evidence toward a genetic influence in this interaction. High resonance magnetic resonance imaging provides a promising efficacious avenue in future mouse model hearing loss investigations.

## Introduction

Hearing loss is the most common human sensory deficit worldwide and age-related hearing loss has now been associated with cognitive decline and increased risk of incident dementia and depression (Loughrey et al., 2018; Choi et al., 2021). The relationships and implication of changes in auditory pathways and in the volumes of cortical and subcortical regions as a function of age is not yet well understood but is a growing nascent area of research. Recent studies using imaging-based quantitative analysis of brain structures have uncovered a connection between hearing loss and decreased volume of auditory regions as well as a biased decline in right temporal lobe volume (Lin et al., 2014). However, work in this area is difficult due to significant variation in both the environments and genetics of human populations. As a result, large numbers of participants are required to perform well powered investigations on the effect of aging on the human auditory system (Wright et al., 2002).

Generally well conserved genomics between humans and mice make the laboratory mouse an appropriate model in hearing research. We have used a family of genetically diverse strains of mice that mimic the diversity of humans but for which the environment can be controlled. The particular BXD family that we have utilized previously segregates for about 6 million common variants. Both the BXD family and the Hybrid Mouse Diversity Panel superset of strains have driven advances in systems genetics in many research disciplines (Ashbrook et al., 2019; Ohlemiller, 2019). Analysis of these isogenic but diverse cohorts allows control of both genetic and environmental factors and can be extended to multiple time points in development and aging; as such, it is possible to study gene-by-environment effects in manners that have high translational relevance to humans. While the mouse brain has less relative white matter than that of human, it contains a similar range of cell sizes and axonal diameters and likely provides an excellent translational model for imaging studies (Johnson et al., 2019).

Despite these advantages we currently do not known much about age-related changes in auditory system anatomy as a function of genotype. Conventional neuroanatomical methods rely on imaging of histological sections, and this poses difficulty in accurate estimation of the true volume and morphology of key components of the auditory system. Despite magnetic resonance imaging (MRI) of the human brain being a mainstay in clinical care and neuroscience research, technical difficulties of acquiring images in the mouse with sufficient resolution has limited research advances. Achieving sufficient power and precision for the quantitative analysis of structural variation to demonstrate relationships with behavior is difficult; however, it will ultimately be necessary to link neuroanatomical and molecular variation in brain to the key function and behavioral differences in audition.

In recent years, advances in technology have allowed systematic analysis of mouse central brain structures by structural and functional MRI (Wang et al., 2020). Johnson et al. (2022) have developed a process to efficiently generate magnetic resonance histology (MRH) images of the mouse brain with comprehensive 3D volumetric labeling (Johnson et al., 2022). Their results proved generalizable to different ages, sexes, and genotypes with spatial resolution more than 500,000 times that of comparable clinical protocols. This methodology generates 3 dimensional images without appreciable distortion and allows acquisition of hundreds of specimens a year. This is sufficiently high to target neurogenetic and genome-wide mapping studies and enable global analysis and genetic dissection of variation in CNS architecture with mice which require large numbers of specimens.

This process has been used in global brain analysis; however, analyzing an individual system utilizing this methodology has not been done (Wang et al., 2020). No three-dimensional mapping of the auditory pathway in mice is available. The objective of this study was to perform a volumetric analysis on the auditory pathway structures in mice and to evaluate possible influences of genetics and aging. We aim to do so by leveraging the MRH data set. Findings can potentially lay foundations for the neuroanatomical mechanistic basis in the findings observed in auditory research and provide novel avenues into future genetics research.

## Materials and methods

### Mice

Mouse experiments were completed in accordance with Duke University Institutional Animal Care and Use Committee. Here we have studied 12 members of the BXD family obtained from University of Tennessee Health Science Center: BXD24, BXD29, BXD34, BXD43, BXD44, BXD48a, BXD51, BXD60, BXD62, BXD65b, BXD89, and BXD101. Upon arrival, animals were housed for at least 10 days to allow adjustment to a new environment. Mice were housed in HEPA-filtered cages with standard, commercial lab bedding either individually or in pairs (Wang et al., 2018, Wang et al., 2020). They were fed a standard laboratory chow *ad libitum* diet and maintained a 12-h light/dark cycle and were subsequently aged. Two cohorts of mice were imaged. Mice of all 12 BXD strains were aged to either 3 months of age, categorized as young mice, or to fifteen months, categorized as old mice. Upon aging, the mice were sacrificed, and their brains fixed, imaged, and analyzed. Johnson et al. (2022) described the process of tissue preparation, imaging, analysis, and validation of accurately aligned multimodal 3D images of the mouse brain with the high-dimensional integrated volume with registration (HiDiver) suite of methods (Johnson et al., 2022). This is summarized in the following sections “Tissue preparation” and “Image acquisition and analysis.” For each mouse undergoing the imaging protocol, sex, age in days, and body weight in grams were also obtained.

### Tissue preparation

Animals were anesthetized with Nembutal (75 mg/kg) and a catheter was inserted into the left ventricle of the heart. A mixture of 0.9% saline and ProHance (10:1) followed by a mixture of 10% buffered formalin and ProHance (10:1) were perfused with a peristaltic pump. ProHance is a chelated gadolinium compound (Gd, a transition metal with unpaired electrons) commonly used in clinical MRI as a contrast agent and reduces the spin lattice relaxation time (T1) (Johnson et al., 2007; Wang et al., 2020). Following this, the skull was removed and placed in 10% buffered formalin at 4°C for 24 h and the mandible was removed to enable use of a smaller radiofrequency coil. Following fixation, the brain was kept in 1% Prohance/saline solution for at least 3 weeks to allow for adequate rehydration. This allowed for a reduction of T1 to ∼ 100 ms and T2 to ∼ 25 ms. Fixation reduced the apparent diffusion coefficient by ∼ 4X and so b values were increased relative to *in vivo* studies. Specimens were mounted in a 12-mm-diameter plastic cylinder filled with fomblin, an inert fluorocarbon that reduces susceptibility artifacts (Wang et al., 2020).

### Image acquisition and analysis

Specimens were mounted in a 12 mm diameter radiofrequency (rf) coil constructed from a single sheet of silver foil which yields a low resistance. To tune the coil, necessary capacitance is added by placing a layer of dielectric material at the juncture of the coil ends. The resulting unloaded *Q* is > 500. MRH images were acquired on a 9.4T vertical bore Oxford magnet with Resonance Research gradients providing peak gradients of 2,000 mT/m. The scanner was controlled by an Agilent Direct Drive console with VnmrJ 4.0 software. Three-dimensional (3D) diffusion weighted images were acquired with a Stejskal Tanner rf refocused spin echo sequence with TR/TE of 100/12.7 ms and b values of 4,000 s/mm^2^. Forty-six 3D volumes, each with a different gradient angle along with five baseline (b0) images distributed throughout the four-dimensional (4D) acquisition, were acquired. Sampling angles were uniformly distributed on the unit sphere. Compressed sensing was used with 8X acceleration to reduce the acquisition time to 11.7 h/specimen. This resulted in a 4D image array with isotropic spatial resolution of 45 μm (voxel volume of 91 pl) (Wang et al., 2020).

The 4D array (256 × 256 × 420 × 51) was created by first averaging the five b0 images. To correct for eddy current distortion, the diffusion-weighted 3D volumes were then registered to this template. The 4D array was processed with DSI Studio yielding the following scalar images: axial diffusivity (AD), radial diffusivity (RD), mean diffusivity (MD), fractional anisotropy (FA), and color fractional anisotropy (ClrFA) using the DTI model (Yeh et al., 2021). A Matlab script averaged the diffusion weighted images to yield a DWI volume.

A 3D label set consistent with the Allen Brain Atlas Common Coordinate Framework version 3 (CCFv3) was registered to all the scalar images (Johnson et al., 2022). The CCFv3 consists of 461 labels. A reduced but comprehensive subset of 180 ROIs (per hemisphere) that combine smaller adjacent sub-volumes was generated as many ROIs were too small to be reliably registered. A symmetric atlas was generated by reflecting the label set through the midline to minimize bias in lateral comparisons (Bowden et al., 2011). This label set of 180 total ROIs in each half of the brain was used to provide a full and isotropic parcellation of each brain (Johnson et al., 2010; Calabrese et al., 2015).

### Auditory pathway region of interest selection

**FIGURE 1:**
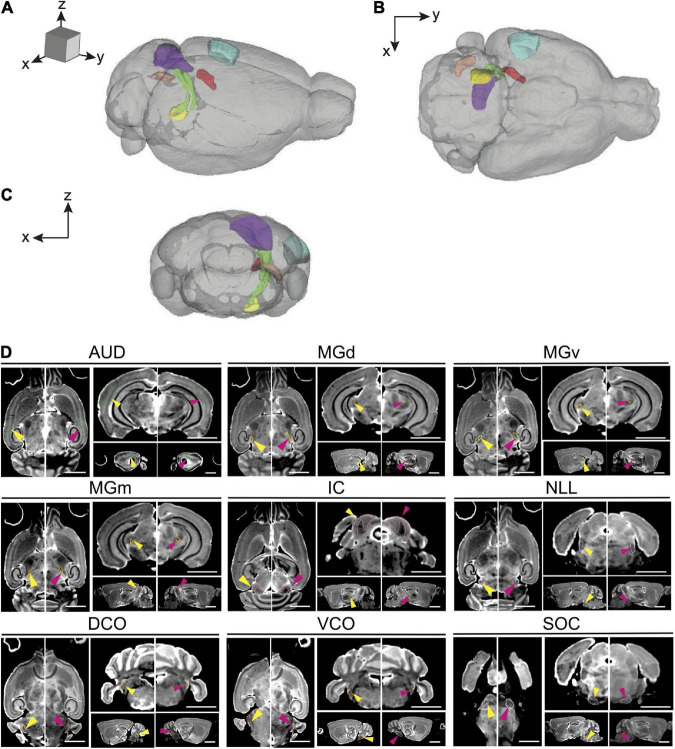
Magnetic resonance imaging of the auditory pathway. **(A–C)** 3D volume rendering of mouse auditory brain structures of the average old mouse structure size within the **(A)** sagittal, **(B)** axial, and **(C)** coronal planes. Colored structures represent specific central auditory structures: Blue; auditory cortex, Red; medial geniculate complex (dorsal, ventral and medial), Purple; inferior colliculus and its nucleus, Yellow; superior olivary complex, Orange; dorsal cochlear nucleus, Green; lateral lemniscus. **(D)** Representative axial, coronal, and sagittal plane magnetic resonance images of all structures in the auditory pathway of a young and an old BXD51 mouse. The images have an arrow aimed at the individual outlined structure. Within each image pair, the left image with a yellow arrow is the young mouse image and the right image with a pink arrow is the old mouse image. Scale bars in all planes is 2.5 mm. AUD, Auditory cortex; MGd, Medial geniculate nucleus, dorsal part; MGv, Medial geniculate nucleus, ventral part; MGm, Medial geniculate nucleus, medial part; IC, Inferior colliculus; LL, Lateral lemniscus; SOC, Superior olivary complex; DCO, Dorsal cochlear nucleus; VCO, Ventral cochlear nucleus.

The central auditory system is comprised of a complex set of nuclei extending from the cochlear nuclei up to auditory cortical regions. The largest of these structures include, in ascending order, the cochlear nuclei, the superior olivary complex, tract and nuclei of the lateral lemniscus, the inferior colliculus, and the medial geniculate nuclei. The spatial mapping which originally defined a set of 180 regions of interest (ROIs) per side was pared down to a subset of 11 well defined ROIs that are part of the auditory system. The three subdivisions of the medial geniculate nucleus were summed and combined as a separate summation ROI which was included in addition to its individual subdivisions, totaling 12 ROIs included in analyses. These are, with abbreviations, as follows (Figure 1):

1. Auditory cortex (AUD)
2. Medial geniculate nucleus (MGN)
3. Medial geniculate nucleus, dorsal part (MGd)
4. Medial geniculate nucleus, ventral part (MGv)
5. Medial geniculate nucleus, medial part (MGm)
6. Inferior colliculus (IC)
7. Inferior colliculus, central nucleus (Icc)
8. Lateral lemniscus (LL)
9. Lateral lemniscus, nucleus (NLL)
10. Superior olivary complex (SOC)
11. Dorsal cochlear nucleus (DCO)
12. Ventral cochlear nucleus (VCO)

Total brain volume (TBV) was also measured and used to determine the selective of effects on auditory ROIs.

### Voxel-based volumetric analysis

Utilizing the 12 ROIs as described in section “Auditory pathway region of interest selection,” a voxel-based volumetric analysis was completed with the primary aim of understanding relationships in auditory pathway structure volume. The imaging protocol provided a voxel volume of 9.1 × 10^–5^ mm^3^ per voxel and, utilizing this factor, the voxel count of each ROI was converted to volume. The size of the two individual structures in each mouse brain from the left and right hemispheres was summed for an overall structure volume in the entire mouse brain. This volume was averaged in each age group and strain pairing for analysis of the aging and genetic basis in auditory structure size. We then calculated within-animal coefficient of variation for all structures to assess precision and segmentation repeatability of our measurements.

For quantitative analysis, we calculated percentage volumes relative to whole brain (or formally “whole brain volume minus ROI”) to factor out global or non-selective changes in brain volume. We also used a linear regression model to determine age-related structural changes controlling for body weight, sex and total brain volume and potential confounding factors. This statistical approach was adapted from Williams (2000), in which the rationale behind statistical treatment of phenotypic data and linear regression methods phenotypic is explained in greater detail (Williams, 2000). We first confirmed data assumptions to conduct linear regression, including linearity and normality. In the model, we regressed individual structural volume on various mice characteristics including age in months, weight in grams, sex, and total brain volume. Akaike information criterion was used for parsimonious model criteria selection. In the end, sex was not included in the model because this variable was not a significant covariate for any auditory system ROI. Associations were considered significant if the confidence intervals at an alpha = 0.05 threshold did not overlap with 0.

### Statistical analysis

All data processing and statistical analysis were performed with the R 4.1.1 statistical computing software (R Core Team, 2021). We utilized the tidyverse collection of packages: in particular dplyr 1.0.7, tidyr 1.1.3, tibble 3.1.3 for data manipulation, including calculation of means and standard errors, and analysis, including calculation one-way paired analysis of variance with corresponding *p*-values as well as creation of linear regression models; and ggplot2 3.3.5 for creation of figures (Wickham et al., 2019). Due to the greater number of old mice data, pair analyses matched unpaired old mice with an already paired isogenic young mice that were similar in sex and weight.

## Results

104 mice were imaged with the protocol in sections “Tissue preparation”-“Image acquisition and analysis.” Young mice (*n* = 45) had a median age of 98 days, ranging between 72 and 129 days, and a mean (SD) weight of 22.8 (4.7) grams. Old mice (*n* = 59) had a median age of 446 days, ranging between 341 and 687 days and a mean weight of 29.7 (6.1) grams. There was a slight female preponderance in sex distribution with 45 male mice and 59 female mice (43.3% male), within which there were 23 male and 27 female mice (46.0% male) in the young cohort and 22 male and 32 female mice (37.3% male) in the old cohort. Representative 3D volume rendering and magnetic resonance images of the structures in the auditory pathway are depicted in Figure 1. A summary of the mean volumes of all 12 ROIs as well as TBV is tabulated in Table 1. Raw data for all 104 mice is presented and available in Supplementary material.

**TABLE 1:** Summary volumes of region of interests.

	Mean volume, mm^3^ (SD)		
Structure	Young (*n* = 45)	Old (*n* = 59)	*P*-value
TBV	416 (21)	433 (23)	< 0.001
AUD	4.67 (0.35)	4.43 (0.31)	< 0.001
MGd	0.142 (0.018)	0.148 (0.017)	0.003
MGv	0.247 (0.025)	0.253 (0.024)	0.002
MGm	0.211 (0.014)	0.226 (0.016)	< 0.001
MGN	0.600 (0.054)	0.627 (0.052)	< 0.001
IC	3.28 (0.28)	3.54 (0.33)	< 0.001
Icc	0.964 (0.077)	1.03 (0.21)	0.005
LL	0.724 (0.055)	0.782 (0.055)	< 0.001
NLL	0.669 (0.062)	0.744 (0.055)	< 0.001
SOC	0.654 (0.061)	0.699 (0.067)	< 0.001
DCO	0.482 (0.037)	0.507 (0.042)	< 0.001
VCO	0.737 (0.058)	0.755 (0.058)	0.004

Mean volume of all regions of interest in the auditory pathway investigated. A significant difference was noted between young and old mice in every structure analyzed through paired Student’s *t*-test. The higher number of old mice were paired with a matched isogenic young mouse by sex. TBV, Total brian volume; AUD, Auditory cortex; MGd, Medial geniculate nucleus, dorsal part; MGv, Medial geniculate nucleus, ventral part; MGm, Medial geniculate nucleus, medial part; MGN, Medial geniculate nucleus; IC, Inferior colliculus; Icc, Medial geniculate nucleus, medial part; LL, Lateral lemniscus; NLL, Lateral lemniscus, nucleus; SOC, Superior olivary complex; DCO, Dorsal cochlear nucleus; VCO, Ventral cochlear nucleus.

### Volumetric differences arise between strains

**FIGURE 2:**
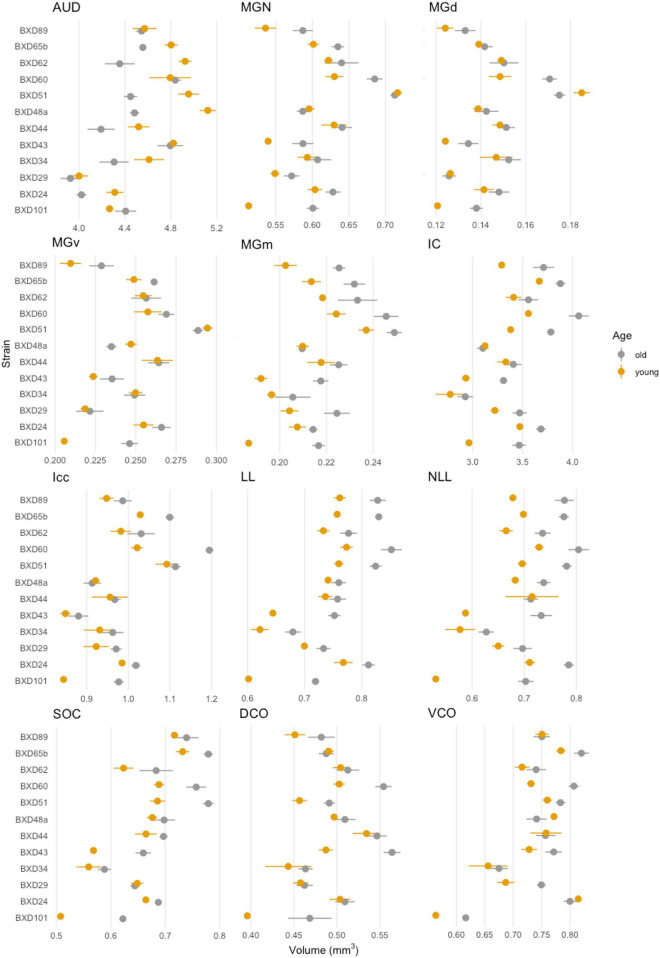
Summary of individual structure volumes by strain. Mean individual structure volume with horizontal lines indicating standard error in all strains. A variety of strain-based differences in both absolute volume and age-related change is observed in all structures. A range of 4–6 mice were in each age cohort and strain pairing with the exception of BXD101, in which only one mouse was imaged in the young group. AUD, Auditory cortex; MGd, Medial geniculate nucleus, dorsal part; MGv, Medial geniculate nucleus, ventral part; MGm, Medial geniculate nucleus, medial part; MGN, Medial geniculate nucleus; IC, Inferior colliculus; Icc, Inferior colliculus, central nucleus; LL, Lateral lemniscus; NLL, Lateral lemniscus, nucleus; SOC, Superior olivary complex; DCO, Dorsal cochlear nucleus; VCO, Ventral cochlear nucleus.

We first evaluated the absolute volume of the 12 auditory ROIs and the effect of age, sex, and strain. We compiled mean absolute volume in all 12 evaluated auditory structures by BXD strains and mouse age cohort (Figure 2). Although isogenic mice overall had little to no variation in individual structure volume, mean individual structure volumes differed markedly across strains; analysis of variance supported that there are significant variations between groups of mouse strains and auditory structures independent of aging (*p* < 0.0001 for all individual structures). Visualization of these mean values between young and old cohorts do show differences in the effect of aging on volume not only among all structures but also between the various strains evaluated, supporting a genetic influence on the age-related effect on structure volume in the auditory pathway. Interestingly, we observed no intersex differences in both univariate and multivariate analyses between the entire cohort. Coefficient of variation within-animal between hemispheres were low in all structures, ranging between a minimum 1.64% ± 0.01% SEM in the inferior colliculus and a maximum 4.04% ± 0.04% SEM in the dorsal cochlear nucleus, supporting reproducibility of our imaging and parcellation methodology.

### Aging affects auditory pathway structure size heterogeneously

**FIGURE 3:**
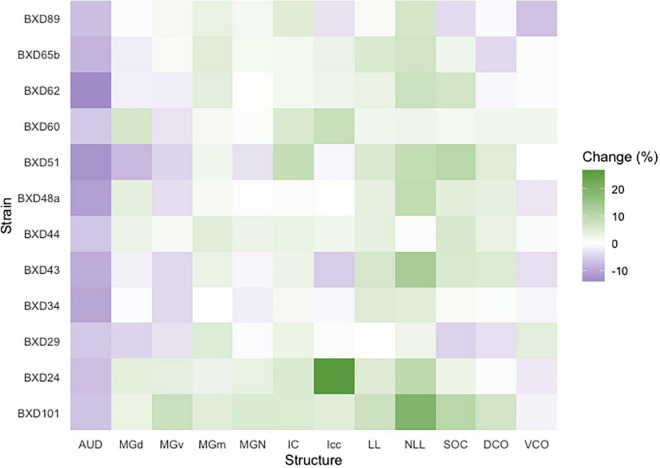
Brain proportion percentage change with aging. A heatmap matrix showing percentage change in individual structure brain proportion with age in every strain evaluated. Except for the auditory cortex in which there was an age-related decline in brain proportion, there were substantial strain-based variation in all structures with aging. A range of 4–6 mice were in each age cohort and strain pairing with the exception of BXD101, in which only one mouse was imaged in the young group. AUD, Auditory cortex; MGd, Medial geniculate nucleus, dorsal part; MGv, Medial geniculate nucleus, ventral part; MGm, Medial geniculate nucleus, medial part; MGN, Medial geniculate nucleus; IC, Inferior colliculus; Icc, Inferior colliculus, central nucleus; LL, Lateral lemniscus; NLL, Lateral lemniscus, nucleus; SOC, Superior olivary complex; DCO, Dorsal cochlear nucleus; VCO, Ventral cochlear nucleus.

We then quantified the age- and strain-/genetic-based relationship and interaction with auditory structure volume. Among all strains, a significant change in absolute structure volume was observed in all individual structures evaluated as well as total brain volume (Table 1). Utilizing percentage change in the proportion of total brain volume occupied for each structure between young and old mice of the same strain, we observe that 97/144 (67.4%) of structure-strain pairings in the auditory pathway demonstrated an increase in size after aging, to varying degrees (Figure 3). Of the minority of structure-strain pairings that decreased in size with age, the auditory cortex (AUD) brain volume proportion decreased with age in all strains, to varying degrees. The remainder of the 35 pairings that demonstrated a decrease in structure volume with aging were heterogeneously distributed between all remainder structures and strains, except for the medial portion of the medial geniculate nucleus which displayed an increased in brain volume proportion with age.

**FIGURE 4:**
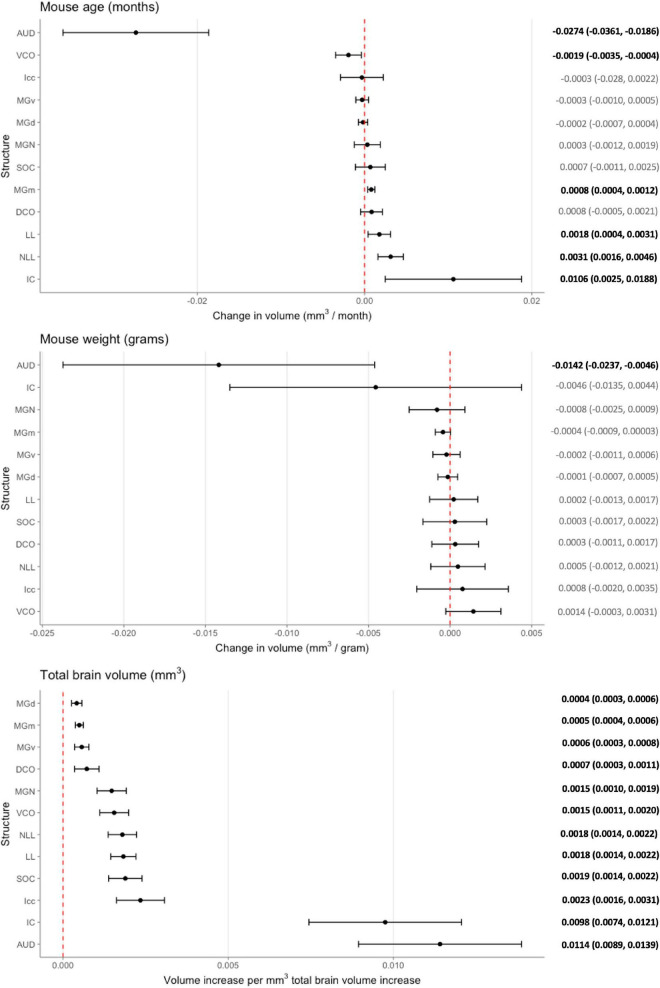
Multivariable analysis of the association of mouse age, weight, and total brain volume on auditory structure volume. Markers indicate degree of individual structure change per month mouse age **(top)**, gram mouse weight **(middle)**, and mm^3^ total brain volume **(bottom)**, respectively. Horizontal lines indicate 95% confidence intervals. Raw coefficient values with their 95% confidence intervals were listed on the right with bolded values indicating significance. Structures were arranged from the lowest to greatest coefficient in each chart. AUD, Auditory cortex; MGd, Medial geniculate nucleus, dorsal part; MGv, Medial geniculate nucleus, ventral part; MGm; Medial geniculate nucleus, medial part; MGN, Medial geniculate nucleus; IC, Inferior colliculus; Icc, Inferior colliculus, central nucleus; LL, Lateral lemniscus; NLL, Lateral lemniscus, nucleus; SOC, Superior olivary complex; DCO, Dorsal cochlear nucleus; VCO, Ventral cochlear nucleus.

While these initial findings do support genetic variation’s influence on structure volume, interpretation is limited as they do not account for possible confounders. We created a voxel-based multivariable linear regression model to control for such factors including total brain volume and mouse weight in the age and structure volume relationship (Figure 4). Six of the 12 ROIs evaluated in our multivariable analysis demonstrated a significant age-related effect independent of global brain volume or mouse weight changes. In ascending rate of volume change with age, these six structures were the auditory cortex [change in volume (95% confidence interval): –0.0274 (–0.0361, –0.0186) mm^3^/month], ventral cochlear nucleus [–0.0019 (–0.0035, –0.0004) mm^3^/month], medial division of the medial geniculate nucleus [0.0008 (0.0004, 0.0012) mm^3^/month], lateral lemniscus [0.0018 (0.0004, 0.0031) mm^3^/month], the nucleus of the lateral lemniscus [0.0031 (0.0016, 0.0046) mm^3^/month], and the inferior colliculus [0.0106 (0.0025, 0.0188) mm^3^/month].

To evaluate the model, adjusted R2 values were utilized to estimate corrected goodness-of-fit. The model only accounted for a low amount of the variation of structure volume in three of the 12 structures: dorsal division of the medial geniculate nucleus (24.8%), ventral division of the medial geniculate nucleus (23.8%), and central nucleus of the inferior colliculus (14.6%). However, it did account for a high amount of variation for the remaining ROIs, ranging from 36.1% in the whole medial geniculate nucleus to 63.5% in the lateral lemniscus. A significant portion of the remaining variation is likely accounted for by genetic differences, which our model does not account for due to a relatively low number of mice in each strain and age pairing.

### Mouse weight is negatively associated with auditory cortex volume

While our analysis focused on the effect of age on auditory structure volume, we did observe notable findings in the other covariates in our model (Figure 4). We observed that the auditory cortex is the sole ROI that displays a significant relationship with mouse weight that is age and total brain volume independent. For every gram increase in mouse weight, a corresponding 0.0142 mm^3^ decrease in auditory cortex volume is observed. Expectedly, all individual structure volumes evaluated do increase commensurately with increasing total brain volume (Figure 4). Unsurprisingly, the corresponding magnitude of the ratio between volume increases does align with the absolute size of the structure itself: for example, the large auditory cortex demonstrates a 0.0114 mm^3^ increase in size per 1 mm^3^ total brain volume, whereas the small dorsal cochlear nucleus, commensurate to its relatively smaller size, demonstrates a 0.0007 mm^3^ increase per 1 mm^3^ total brain volume.

## Discussion

In this study, we leverage MRI data sets of a genetically diverse set of mouse strains in a comprehensive quantitative analysis of the mouse brain auditory pathway cytoarchitecture (Johnson et al., 2022). We observed strain-based volumetric differences in all individual structures of the auditory pathway as well as a heterogenous effect of aging in different strains upon structure size. Secondarily, we describe an independent negative effect of mice weight on auditory cortex volume. Our results support a genetic basis and influence in auditory structure volume and identify age-related volumetric changes in auditory structures. This study, to our knowledge, was the first attempt to utilize magnetic resonance histology for the volumetric analysis of a single functional pathway in the mouse model.

### Imaging and parcellation of the auditory pathway

Current knowledge of the neuroanatomical basis of the auditory pathway is limited, and it is difficult to derive conclusive inferences from available work as with no single method is sufficient to provide a complete picture of the brain. The mouse brain is ∼ 3,000 times smaller than the human brain requiring a commensurate increase in spatial resolution for comparable anatomic measurement. Many advances in the molecular and neuroimaging techniques used in the study of fine structures have led to revelations in neurosciences research. Investigations into this area are becoming possible with technological advances and this field is becoming a nascent frontier for the future of research.

The high-resolution MRH and registration algorithm described in this study rely on extension of previous methods and merging of histological methods that allows collection and registration of key structures in whole individual brains in an efficient and systematic method. This process defines features accurately and without the registration issues that compromises many histological procedures. Utilizing this, we have successfully imaged, identified, and registered all individual structures of the auditory pathway. In addition, individual nuclei of the lateral lemniscus and inferior colliculus as well as the three subdivisions of the medial geniculate nucleus were able to be parcellated from the whole structure. The voxel volume at 9.1 × 10–5 mm^3^ per voxel is more than 21,000 times smaller than the voxels of the Human Connectome Project and nearly 90,000 times smaller than routine clinical exams (Uğurbil et al., 2013; Van Essen et al., 2013). While much of the previous work in small animal imaging research has been focused on achieving greater spatial resolution, this more recent work has focused on increasing throughput. This high throughput fulfills large power requirements needed for quantitively genetic dissection investigations targeted at neurogenetic and genome-wide mapping. Imaging studies have shown evidence of homology between human and mice (Keifer et al., 2015). There is great translational potential in these novel approaches for understanding genetic variation and environmental influence that underlie differences in hearing behaviors across species. Further advances in this technique toward artificial intelligence-driven workflows and algorithms can allow complex modeling and elucidation of the genetic and environmental influences of possible causal relationships between cellular and subcellular structure and functional behavior (Johnson et al., 2022).

### Strain-based volumetric differences

Advanced neuroimaging efforts combined with classical genetics and genome-wide association studies such as the Human Connectome Project represent the frontier for *in vivo* understanding of the neural connections of individuals (Van Essen et al., 2013). However, there is little conclusive evidence in the neuroanatomical basis in hearing. Not only is the variability of the absolute and relative size of the individual brain regions highly diverse within populations, there is also wide heterogeneity in the brain alterations found in hearing loss (Andrews et al., 1997; Kral et al., 2016; Tarabichi et al., 2018). Studies are also regularly limited by high degrees of measurement inconsistency. With the progress of small animal neuroimaging as described, there is enormous potential for utilization in genetics-based research as it avoids many of these issues by virtue of a controlled environment. In our analysis, we observed significant strain-based differences in all auditory structures analyzed in both age cohorts, supporting a genetic disposition to both structure volume and the aging effect within the mouse (Table 1 and Figure 1). There is evidence currently supporting heritability in all imaging scalar metrics; however, mapping of the volumetric phenotypic trait requires the imaging data of significantly more strains than was analyzed in this study—approximately to the degree of 600 mice. The throughput of this imaging and processing methodology makes this a reality. Complex interactions between variants as well as complex environmental factors and confounders can very soon be evaluated and understood. More realistic and robust population models that incorporate levels of genetic variation comparable to human populations are now possible.

### Mixed effects of aging on structure volume

Hearing is one of our main sensory senses and presbycusis is the most common sensory deficit in the elderly with severe social and health implications (Lin et al., 2011; Goman et al., 2017). Age-related hearing loss or presbycusis is the most prevalent sensory deficit in the elderly population (Huang and Tang, 2010). It is known that the aging process is associated with significant decrease in global and regional brain volumes, particularly in the temporal lobe and temporal gyri that comprise the human auditory cortex, and that hearing impairment is associated with acceleration of this brain volume decline (Scahill et al., 2003; Lin et al., 2014). These age-associated structural changes is a potential mechanism underlying the association between hearing impairment and cognitive decline and dementia and a better understanding of this process may help to elucidate expected age-related changes from neurodegenerative pathology (Lin and Albert, 2014). Our results are consistent with clinical findings in the auditory cortex and ventral cochlear nucleus, showing a decline in size with aging independent of global volume changes (Frisina and Walton, 2006). The most pronounced change with aging was observed in the auditory cortex, to a greater degree than that accounted for it being the largest structure in the pathway. The mechanistic basis for such findings has not been conclusive. In addition to gray matter loss and age-related atrophy of these areas, it has been proposed that a ventricle expansion effect contributes to changes in morphology (Eckert et al., 2019; Koops et al., 2020). The basis for the relationship between volume declines and behavior in hearing impairment is also not clear; hypotheses diverge as to whether auditory deprivation lead to declines in auditory area or if aging-caused changes in the peripheral and central auditory structures lead to subsequent hearing loss (Ren et al., 2018; Eckert et al., 2019).

In direct contrast, age-associated volume increases were observed in the medial division of the medial geniculate nucleus, the lateral lemniscus and its nucleus, and the inferior colliculus. While the age-associated changes reported in the cochlear nucleus and the auditory cortex have been a focus of research, these other structures in the auditory pathway are less studied (Tarabichi et al., 2018). A degree of increase in size is expected as the brains of some strains of mice grow in size as a function of age even in advanced age; however, our model does control for this factor (Peirce et al., 2003). While neurogenesis has been known in certain areas of the mouse brain even into late adulthood, such as the olfactory bulb via the rostral migratory stream, studies reflecting such a concept in the auditory system are few (Williams et al., 2001). Upregulation of parvalbumin expressing neurons in the inferior colliculus and medial geniculate body has been reported and is likely related to changes in frequency processing (Ouda et al., 2008; Gray et al., 2013; Engle et al., 2014). There have also been findings of age-related compensatory changes in certain auditory neuronal populations to maintain information fidelity; similarly, projections to the auditory cortex from the medial geniculate nucleus that remain specific with age have been found and likely have a gain control function (Engle et al., 2014; Gray et al., 2014; Chen et al., 2019). These support the hypothesis of a compensatory increase in auditory structure volume as a response to auditory cortex decline. Alternatively, the increase in size of these structures may be a result of recruitment by other systems after auditory deprivation; such an effect has been observed in humans and hypothesized to support speech perception by the visual system after hearing impairment (Slade et al., 2020; Manno et al., 2021).

### Mouse weight displays negative association with auditory cortex volume

Beyond age and strain-based changes, we also observed a significant independent negative relationship between mouse weight and the auditory cortex. To the best of our knowledge, this volumetric relationship has not been reported in literature. There is a hypothesized relationship between obesity and hyperlipidemia with hearing degeneration via an oxidative stress-based inflammatory response in the peripheral neurons (Hwang et al., 2013). Our study suggests that these same factors may have an influence in the cortical structures. Additionally, the vascular implications of obesity may also play a role as it is known to have a vasoconstrictive effect on the vascularly sensitive inner ear that may subsequently influence higher order structures (Dhanda and Taheri, 2017).

### Limitations

There are several limitations of this study. While we aimed to age mice to certain time points considered young and old, there was a significant age range in both cohorts of the study which we attempted to control via continuous modeling. The low number of strains evaluated in this study limited potential evaluation of genetic differences and modeling was not able to incorporate strain-based differences. Imaging of an expanded set of strains is ongoing and examination of volumetric differences using complex trait analysis will soon be possible with the aim to localize genetic factors modulating structural variation. Despite these limitations, our study remains one of the few to attempt structural quantitative analysis in the mouse model of a specific functional pathway. The great majority of studies have focused on behavioral variants with a rationalistic basis anchoring on an underlying mechanism in the brain; however, a thorough understanding of the neuroanatomical structural relationships and underpinnings is ultimately necessary.

## Conclusion

We present a novel approach for the volumetric analysis of the auditory pathway in the mouse utilizing magnetic resonance histology. Our results support a genetic basis and influence in auditory structure volume and identify novel age-related volumetric changes in auditory structures. An understanding of the structural differences in auditory structures is crucial in unwinding the genetic groundworks and links of hearing and is ultimately foundational knowledge in the pursuit of understanding hearing physiology and impairment. Robust structural analyses within individual functional systems will allow novel means of translation integral in our understanding of the inner workings in the complex brain.

## Data availability statement

The original contributions presented in the study are included in the article/Supplementary material, further inquiries can be directed to the corresponding author/s.

## Ethics statement

The animal study was reviewed and approved by the Duke University Institutional Animal Care and Use Committee.

## Author contributions

ED, BO, YN, RW, GJ, and RF contributed to conception and design of the study. GC, JC, KH, YQ, and GJ contributed to novel technique development, data collection and curation, and database organization. ED and RW performed the statistical analysis and interpretation. ED and BO wrote the first draft of the manuscript. YN and GJ wrote sections of the manuscript. RF, RW, and GJ contributed to critical manuscript revision. RF contributed to funding acquisition and project supervision. All authors contributed read, and approved the submitted version.

## Supplementary material

Supplementary Table 1 Complete raw volumetric data using magnetic resonance imaging for 104 mice for 11 well defined regions of interests as well as total brain volume separated by hemisphere. Mice strain, sex, weight in grams, age in days, and age category (young/old) were included and the data set is currently sorted by strain and age category. Within analysis, the three subdivisions of the medial geniculate nucleus were summed and combined as a separate summation ROI not included in this raw data.
